# Rare Psychopathology Associated with Progressive Supranuclear Palsy (PSP) with Frontotemporal Dementia (FTD) Phenotype - A Case Report

**DOI:** 10.1192/j.eurpsy.2025.2047

**Published:** 2025-08-26

**Authors:** S. Garg, O. Afroz, V. Patil

**Affiliations:** 1 Psychiatry, All India Institute of Medical Science, New Delhi, India

## Abstract

**Introduction:**

Progressive supranuclear palsy (PSP) is a neurodegenerative disorder characterised by supranuclear ophthalmoplegia (SNO), parkinsonism, and postural instability. Overlap with frontotemporal dementia (FTD) has been suggested, with PSP-FTD considered a specific phenotype. Common psychiatric symptoms include apathy and depression, while hallucinations and delusions are rare. Hallucinatory palinopsia is the persistence or recurrence of vivid visual images after the stimulus has been removed. It results from aberrant activation of visual memory circuits, and, while uncommon, is typically seen in conditions such as strokes, space-occupying lesions and seizures.

**Objectives:**

To present a case highlighting unique psychopathology in a patient with PSP-FTD phenotype.

**Methods:**

Clinical case description and literature review.

**Results:**

An 80-year-old male with a 6-year history of progressive behavioural changes, memory disturbances, and motor dysfunction presented initially with apathy and social withdrawal. Memory impairment and gait difficulties followed, along with irritability, aggression, and hypersexual behaviours like inappropriate touching or gesturing towards family members or masturbating in public. Asymmetric intention tremors (left > right) and stereotypic hand movements developed over time. In the past year, the patient began experiencing visual hallucinations, particularly hallucinatory palinopsia, where he persistently saw objects like lizards or water bottles that had been removed from view. These occurred in clear consciousness, were not perceived by others, and would typically last for about an hour. By a multidisciplinary approach, the possibility of delirium was ruled out. A diagnosis of PSP with FTD phenotype was made based on clinical evaluation, including SNO, and neuroimaging. The patient was started on Syndopa and Donepezil. Psychiatric evaluation revealed high scores in domains of Apathy, Disinhibition, Agitation, and Hallucinations on the Neuropsychiatric Inventory (NPI). Psychoeducation was provided, and Quetiapine 12.5 mg was initiated, leading to mild improvement in behavioural symptoms. The patient remains under regular follow-up with plans for medication optimization and physiotherapy inclusion.

**Conclusions:**

Behavioural symptoms in PSP are prevalent and challenging to manage. This case highlights the importance of distinguishing between apathy and depression, as misdiagnosis can lead to unnecessary antidepressant use. The patient’s presentation, including disinhibition, hypersexuality, and less commonly reported visual hallucinations, emphasises the need for comprehensive evaluation and a multidisciplinary approach to management in PSP-FTD cases.

**Disclosure of Interest:**

None Declared

